# Multi-family therapy for bulimia nervosa: a qualitative pilot study of adolescent and family members’ experiences

**DOI:** 10.1186/s40337-022-00606-w

**Published:** 2022-07-03

**Authors:** Anabel Escoffié, Natalie Pretorius, Julian Baudinet

**Affiliations:** 1 Institute of Psychiatry, Psychology and Neuroscience (IoPPN), King’s College London, De Crespigny Park, Denmark Hill, London, SE5 8AZ UK; 2 Maudsley Centre for Child and Adolescent Eating Disorders (MCCAED), Maudsley Hospital, De Crespigny Park, Denmark Hill, London, SE5 8AZ UK

**Keywords:** Adolescent, Bulimia nervosa, Eating disorders, Multi-family therapy (MFT), Family therapy for bulimia nervosa (FT-BN), Family based treatment (FBT)

## Abstract

**Background:**

Multi-family therapy (MFT-BN) is a new treatment for adolescent bulimia nervosa with emerging empirical support. It extends the bulimia nervosa focussed family therapy model, by offering treatment in a group setting. Up to nine families work together with a team of clinicians over the course of 20 weeks. No qualitative study to date has investigated the experience of MFT-BN. This study aimed to explore this from the adolescent and parent/caregiver perspective.

**Methods:**

Participants from two consecutive MFT-BN groups facilitated at the Maudsley Hospital in London, UK, were invited to participate in either a focus group or individual qualitative interview about the experience of MFT-BN. Of the 19 eligible participants (from 9 families), 15 (8 parents, 1 older sibling, 6 adolescents) consented and participated. Audio-recordings of interviews and focus groups were transcribed verbatim and analysed using reflexive thematic analysis.

**Results:**

Three main themes were identified; (1) seeing and being seen, (2) holistic shift, (3) the unspoken. Participants reported overall shifts in cognitions, emotions, behaviours, and relationships both individually and within the family as a result of attending MFT-BN. Meeting other families with similar struggles and sharing experiences, skills and learning helped reduce isolation and promote change. There was also a sense from participants that some things did not, or could not, be spoken about in the group context and that more direct and challenging conversations might have been helpful at times.

**Discussion:**

The current study identifies some of the perceived benefits and challenges of MFT-BN. The three themes demonstrate the holistic nature of change that can occur across the treatment, as well as the power and limits of the group therapy setting and process. Further research is needed to explore the experience of MFT-BN and its outcomes across a more diverse range of participants and treatment settings.

## Plain English Summary

Multi-family therapy (MFT-BN) is a new treatment for adolescent bulimia nervosa. It involves a group of families (maximum nine) working together with a team of therapists over the course of 20 weeks. This study aimed to explore the experience of MFT-BN from the adolescents and parent/caregiver perspective, as this has not been explored before. Participants from two consecutive MFT-BN groups were invited to participate in either a focus group or individual interview. 8 parents, 1 older sibling, and 6 adolescents participated. Three themes were identified from what they said. Participants described overall shifts in their thoughts, emotions, behaviours, and relationships both individually and within their families, as a result of attending MFT-BN. Meeting other families with similar struggles and sharing experiences, skills and learning helped reduce isolation and promote change. There was also a sense from participants that some things did not, or could not, be spoken about in the group context and that more direct and challenging conversations might have been helpful at times. More research is needed to explore the experience of MFT-BN and its outcomes across a more diverse range of participants and treatment settings.

## Background

Bulimia nervosa (BN) is a serious mental health disorder that typically develops in adolescence [1–3]. Alongside the main eating disorder symptoms (binge eating and compensatory behaviours, body shape and weight concern/distress), people with BN often experience other co-existing difficulties, such as low mood, self-harm, suicidality, emotion dysregulation and impulsivity [2, 4–9]. Family factors, such as high levels of expressed emotion and parental criticism in response to BN symptoms have also been reported at higher rates compared to parents of young people with anorexia nervosa (AN) or depression [10].

Despite the significant impact of BN on young people and their caregivers, existing research into treatments specifically for young people with BN is limited. BN focussed family therapy (FT-BN) is a recommended treatment for young people in the UK [11], however, outcomes are relatively modest. The NICE (2017) recommendation of FT-BN is largely based on three randomized controlled trials (RCTs) [12–14], in which fewer than 50% of participants were abstinent from bingeing and purging by the end of treatments [12–14]. Clearly, further developments are needed to support both young people with BN and their family members/caregivers.

Multi family therapy (MFT) groups have been developed and used across a number of different physical and mental health disorders for decades [15, 16]. MFT typically involves bringing a group of families together to work with a clinical team over the course of treatment. MFT is usually very activity-based and uses different group constellations (e.g. separated young person, parent and sibling groups, pairs, or mixed groups) to promote sharing and new learning.

In the UK, MFT is now a recommended treatment for adolescents with AN (MFT-AN) [11] and has been manualised for both adolescents [17] and young adults [18]. MFT-AN interventions can be useful as a stand-alone treatment, or as an adjunct treatment alongside other interventions [19]. In the only adolescent outpatient RCT to include MFT-AN, Eisler et al. [20] showed that offering adjunctive MFT-AN alongside family therapy for AN (FT-AN) is associated with better outcomes than FT-AN alone.

Qualitative research into the experience of MFT-AN has identified several benefits afforded from the unique group context [19]. Patients and caregivers describe valuing being able to hear new perspectives, share their own experiences, and learning new skills [21–23]. Nevertheless, MFT can also be described as challenging. Some families have reported concerns about young people mixing in the group and sharing unhelpful eating disorder behaviours, especially if they are at different stages of the illness or recovery [24].

The multi-family group settings may provide additional benefits for adolescents with BN. For example, the group setting might be helpful in providing a supportive and validating context in which to address secrecy and shame (factors often associated with BN), to learn new skills to manage BN and other co-morbid symptoms, and to address family relationships. Young people with BN might also benefit from the social interaction and social support which has been linked to both predisposing and maintenance factors of the illness, and recovery in eating disorders [25, 26].

In an attempt to improve upon outcomes for young people with BN, MFT has recently been adapted specifically for this group (MFT-BN). To date, there has been very little research into MFT interventions for BN; indeed, only one peer review article has been published [19]. In an uncontrolled case series, Stewart et al. [27] found that MFT-BN for adolescents was associated with improvements in bingeing and purging behaviours, co-morbid mood, anxiety and emotional regulation difficulties, and parent/caregiver perceived burden. The authors concluded that the intervention could play an important role as a “first step” towards recovery.

While promising, much more research is needed to replicate these findings and to understand the process of treatment. To begin to address this gap, the current study aimed to explore the qualitative experiences of adolescents and their parents/caregivers of MFT-BN.

## Method

### Participants

A convenience sample of adolescents in treatment at the Maudsley Centre for Child and Adolescent Eating Disorders (MCCAED), a specialist, community based, child and adolescent eating disorder service based at the Maudsley Hospital, UK, was used in this study. Adolescents and their family members were eligible for this study if they were (a) between 13 and 18 years old, (b) had an ICD-10 [28] diagnosis of BN or Atypical-BN, (c) were living at home with at least one parent/caregiver and (d) were currently in treatment at MCCAED. ICD-10 diagnoses were made at assessment with MCCAED during clinical interview with an experienced specialist eating disorder clinician. All participants were approached either face-to-face or via telephone contact to take part in the study by a member of the clinical team. Written informed consent was obtained from all participants who took part in this study. Parental consent was obtained in addition to young person assent for those aged below 16 years.

### Description of the MFT-BN intervention and setting

MFT-BN was adapted from an established MFT-AN model [17]. MFT-BN is based on the same principles as the single-family therapy model for BN [29], but extends it by bringing families together to increase support, understanding, and communication. There is also a strong focus on providing practical skills to manage BN symptoms and related co-morbid difficulties. Each MFT-BN group is closed and consists of four to nine families. Participants are offered a total of 16 × 90-min sessions over the course of 20 weeks. The initial 12 sessions are offered weekly, with the remaining four offered fortnightly. MFT-BN integrates ideas from different therapeutic models, including systemic family therapy, cognitive behavioural therapy and dialectical behaviour therapy. The MFT-BN model has been described in detail elsewhere, including a session-by-session plan [30] and preliminary findings published [27]. Each group varies slightly in content and structure to meet the needs of each specific MFT-BN cohort. The order in which content is delivered is also flexible and can be adapted as needed.

### Study design

Information about the qualitative experiences of MFT-BN was gathered using a combination of focus groups (~ 60 m) and individual interviews (~ 45 m), conducted face-to-face in the clinic be author AE alone. All were semi-structured and followed similar topic guides, that focused on (a) experience and expectations of treatment, (b) reflections on what felt more and less helpful in supporting recovery, (c) the impact of MFT-BN on specific domains (see supplementary material for topic guides). Collecting data in different formats was done intentionally with the aim of increasing data richness [31]. All focus groups and interviews were audio-recorded and transcribed verbatim.

### Data analysis

Transcripts of the interviews and focus groups were analysed using reflexive thematic analysis based on Braun and Clarke’s [32, 33] and Boyatzis’ [34] guidelines. All authors (AE, NP, JB) participated in data analysis and approached it form a critical realist epistemological position, from which meaning and experiences are considered subjective and influenced by social and cultural context. The analysis was more theoretical than inductive as it was driven by FT-BN theory around hypothesised change mechanisms. Themes were identified on a semantic level and the process of analysis was recursive as the researchers went back and forth between the data, codes and candidate themes before arriving at the final findings.

Each data set (i.e. focus groups with parents/caregivers, focus groups with adolescents, interviews with parents/caregivers and interviews with adolescents) was initially analysed independently from the others by author AE. Summaries were then compared to generate initial codes and a codebook for each data set. These were then applied to a sub-sample of the data to check applicability. Codes were then analysed for common overarching themes and sub-themes that could be derived across the different data sets by all three authors (AE, NP, JB). To ensure reliability an independent researcher also reviewed the raw data, initial codes, codebooks and themes at each step of the process. Any discrepancies were resolved through an iterative process, during which the analysing researchers would go back-and-forth between reviewing the data and discussions as a small group about impressions, codes and, eventually, themes. The concept of data saturation was not used during the analysis as it was recognised that different meanings are generated by different researchers and the process is inescapably subjective [35]. No software was used to assist analysis.

## Researcher characteristics

First author AE, an MSc student at the time of data collection, was on clinical placement at MCCAED as part of her family therapy training. NP and JB are both clinical psychologists, trained to the doctoral level. Both work within MCCAED and have extensive clinical experience of working with young people with eating disorders and their families. Both have been involved in developing MFT-AN and MFT-BN treatments and clinically facilitate both frequently. Having mixture of genders (female: AE, NP; male: JB), discipline (family therapy, clinical psychology) and level of experience (MSc: AE, Doctoral: NP, JB) was seen as a benefit in the analysis process, as it provided multiple, unique perspectives and meaning on eating disorders, adolescent development and perspectives on treatments.

## Results

### Sample

Participants from two consecutive MFT-BN groups were invited to participate in this study. The total eligible sample included 19 people (7 mothers, 2 fathers, 1 older sibling, 9 adolescents) from 9 families (group 1, n = 5 families; group 2, n = 4 families). Fifteen (78.9%) of these (6 mothers, 2 fathers, 1 older sibling, 6 adolescents) consented and participated. No specific reasons were provided by those who did not participate. Six of the parents (4 mothers, 2 fathers) and 3 adolescents provided data via focus groups. Separate groups were facilitated for parents/caregivers versus adolescents. Two mothers, one older sibling and three adolescents completed individual interviews.

Adolescents who participated in this study were aged between 13 and 17 at assessment (mean = 15.17, sd = 1.17). Four were diagnosed with BN and two with Atypical BN. Five identified as female and one as male. All were cis gendered. Two participants identified as Asian/mixed Asian, one mixed race, and three as white British. Mean duration of eating difficulties prior to assessment at MCCAED was 15.40 months (sd = 18.26, range = 6–48, median = 7). To ensure anonymity of the sample, no further demographic information was collected for this study. No demographic information beyond gender was collected for parent/caregivers.

### Qualitative responses

Interview data from the focus groups and semi-structured interviews are synthesized and presented below. A total of three themes and five sub-themes were derived (see Table 1).

1. Seeing and being seen

**Table 1:** Themes and sub-themes of adolescent and parent/caregiver experiences

Theme	Subtheme	Illustrative quote
1. Seeing and being seen	1a. Reduced sense of isolation	“It has showed me that other people go through something that I thought was just me. If some people say ´when something happens to me I do this´, I go ´oh my God! that happens to me also´ and its reassuring” (adolescent)
	1b. Learning from and with each other	“It was good listening to other people’s ideas because that helped me think of my own things, of what would help me” (adolescent)
2. Holistic shift	2a. Family connection	“My mum and dad [now] know how to help me, as opposed to just getting angry” (adolescent) “… I am definitely getting along with them [parents] better” (adolescent)
	2b. New insights and coping	“Like making a set plan of the eating, I thought that was helpful ‘cause then like you are not hungry for you to binge or whatever and things like writing down your worries that was helpful” (adolescent) “I think it’s benefited her as I said she has some insight into the reasons why she is doing it, so it’s definitely had a positive impact on her” (parent/caregiver)
	2c. Practical help	“It's a lot of talking obviously, and then there's things like distractions and they do give you a lot of sheets [coping skills leaflets] so you can take stuff home with you so that it doesn't stay at the session cause it's easy to go home and kind of push it to the side but because they give you sheets and then you go through your bag and then you find it and you kind of remember and that's good” (adolescent)
3. The unspoken	[no subtheme]	“[there] weren't exactly rules but … you do start to think ‘well is what I'm about to say, ok’ and stuff like that, so you do kind of have to think twice before you say stuff which really isn't the point I don't think” (adolescent)

A common experience for everyone, regardless of family role, was finding it helpful being with others and working towards a common purpose. Participants described feeling less isolated by being around others struggling with similar difficulties and also benefited from being able to learn and share their own experiences and skills.

- Reduced sense of isolation

Adolescents and parents/caregivers alike described the normalising and validating effects of being in a group context with others who shared the same problem. There was a sense of togetherness within the group, as opposed to feeling lonely in the situation of living with BN.

Parent: “It’s helped for us knowing that somebody else is in the same situation. That makes you feel a lot better knowing that. … I know it’s not nice to say, but that you're not isolated, you're not the only one. That makes you feel ten times better … would recommend that to anyone”

Adolescent: “I think she [parent/caregiver] probably found it extremely helpful that she could speak to other girls my age [with BN] and other parents, you know? Just speaking to other people, so that she doesn’t feel alone in it, as I felt alone in it...” (adolescent)

b. Learning from and with each other

Adolescents and parents/caregivers said that being with others was an opportunity to learn by both talking and listening to other peoples’ points of views. Participants expressed how being in a group context encouraged perspective taking, which enabled everyone to be more receptive and empathic. This process was described as helpful in both understanding others as well as the self.

Adolescent: “It's good to see other people's opinions. It makes your opinion more rounded and you understand and empathise with more people and then you understand more [for yourself]”

Parent: “It gave me a greater understanding of what he [adolescent] was going through because I just didn't understand, I thought what sane person would do that to themselves”

2. Holistic shift

Adolescents and parents both expressed that MFT-BN helped everyone to function and cope better in a myriad of ways. Through different mechanisms, such as learning skills, understanding patterns differently, and having space to think more broadly about general mood and well-being, participants described small and large shifts in cognitions, emotions, behaviours and relationships. Some were small, others large. Some were very obvious and intentional/active changes; others were more about acceptance and slowing down.

- Family connection

Most participants described changes in the way all family members connected as a result of MFT-BN. Participants said that their relationships became closer and more trusting. Others described improvements in communication, as well as in people’s awareness of others’ emotions.

Adolescent: “I think we [mum and I] get on better now, ‘cause like, I think she kind of accepts it now ... accept[s] it in the way that she wouldn't not do anything, but like accepted that this is going on and that having a go at me isn´t gonna work, and she is less in my face about it”

Adolescent: “when we started coming here I think it brought us back closer again ‘cause, like, I’ve got more trust in her”

Parent: “I think our [parent-child] relationship has improved, definitely, we don't argue as much which is good”

b. New insights and coping

While adolescents described multiple shifts across treatment, typically they were more focused on improvements in general wellbeing and coping rather than specific behaviour changes. It was rare for an adolescent to specifically mention BN symptoms. Whereas parents/caregivers often explicitly noted improvements for the adolescents in terms of a reduction in specific bulimic symptoms, such as bingeing, purging and restriction, and a generally healthier approach to food and eating.

Parent: “She [my child] is quite confident now that she can control it [bingeing] and she tells me that she’s been great for quite some time and she is definitely eating much better, and is not a problem for her”

Adolescent: “It’s been really helpful, … its helped, like, [me] cope with things rather than stop eating. ‘Case you can’t really ever do that, I don’t think.”

These improvements were often linked to new insights and understanding around common binge/purge triggers, as well as cognitive, emotional, relational and behavioural patterns associated with symptoms of BN and low mood.

Parent: “I think for them [the adolescents], they learned like their triggers and what makes them feel in that way and then what makes them go in the vicious cycle”

Adolescent: “… normally you don’t think about what’s going on and, like, when you have, like, a binge or whatever, you don’t think about it after so it’s good that we did that to make us see where we want to stop and how we can stop it if that makes sense”

Similarly, several parents/caregivers also specifically spoke about how the adolescents seemed to be managing strong emotions and mood swings more effectively by the end of MFT-BN.

Parent: “I mean, before it was all tears and everything, but now she is much happier, she knows how to look after her body and she wants to set up some type of web that she can help somebody else”

c. Practical help

The focus on skills in the programme was specifically described by many as beneficial. It was often directly linked to improved symptom management, especially related to binge/purge behaviours.

Adolescent: “If I’m feeling anxious a good one for me to do is try and put a lot of sensation in my feet [to ground and distract myself], so like walk on grass or like foot massage ... like walk on a funny texture or something like that”

Parent: “She [adolescent] uses all the techniques that were given to them, you know, to break the cycle [of bingeing and purging] … There are days in which she just you know gives into temptation or whatever, but I think it’s benefited her, she is managing to deal with it better than she did before”

Parent: “On the other hand … in the group as parents, we have been given some skills to cope better in addition too”

3. The unspoken

The final theme identified was related to what remained unspoken in MFT-BN. It related, in part, to different people’s perceptions of the group structure and process, as well as the role that shame and guilt had on what people felt comfortable to discuss.

From the parent/caregiver’s perspective, many reflected that BN symptoms were a difficult subject for adolescents to talk about, especially with their own parents and/or in a group setting. They noted there was something challenging about having to actively addressing emotions such as shame, embarrassment or guilt.

Parent: “… I understand it is a very secretive disorder and, you know, and very embarrassing [to talk about in a group] as well. Especially with all the stigma against it”

From the adolescent perspective, however, several felt the group structure and process inhibited their ability to express themselves at times. Some remarked on avoiding certain topics, or withholding details, particularly around risk issues. This was out of concern that parents or clinicians in the group may ‘overreact’. This balance between openness, confidentiality, and risk felt tricky to navigate for some.

Adolescent: “I think it's quite similar to school, you can tell your teachers some things but there are some things that you know you can't, like things that would put you in danger or whatever but then I'm thinking that's the reason we're here is because we have a disorder and that's putting us in danger, so I didn't really understand that and that made me feel like I had to hold back quite a few things, that I couldn't say everything that I wanted to say because I might be reported or whatever”

Others noted that this could limit how direct or challenging a conversation could be. Some felt more directness was needed sometimes to move the conversations to a more meaningful place. The importance of needing to trust each other’s intentions and not take things too personally was emphasised.

Adolescent: “It was quite cutesy and I think if they were more harsh and I think if we were all more harsh in what we said, then I think a lot more would come out of it and we would think about what other people said a lot more. I think because we were all so careful about what we said and we were all pussyfooting around other people's feelings. I think if we all made a pact at the beginning like what we say, we say not to be horrible or rude or mean it’s just what we are thinking so don’t take it offensively”

To address this, several adolescents expressed a desire for more unstructured time, together as an adolescent group, away from the parents. There was a sense of wanting to share more of their week-to-week experiences in a more informal way.

Adolescent: “I think the structure was good, but I think that there should be a few sessions where nothing has been planned and we literally can sit there and discuss how our weeks have been. I felt sometimes that I just wanted to say if I have had a really crap week. I wanted to tell people how I was feeling and how crap I felt”

## Discussion

The current study aimed to explore the adolescent and the parent/caregiver experience of a 20-week MFT-BN programme. While there is preliminary data suggesting MFT-BN may be an effective intervention for adolescents [27], there is no data, to our knowledge, exploring participant experiences of the treatment.

Overall, three themes were identified from the data; (1) Seeing and being seen (the group as a space for support and learning), (2) holistic shift (a space that facilitated a broad array of changes) and (3) the unspoken (reflections on the scope and limitations of the group process). It was strongly noted by adolescents and parents/caregivers alike that MFT-BN is a space of learning and support. Joining with other families in a similar situation helped participants to feel less isolated, less burdened by the illness and was described as helpful in learning new ways of managing the illness.

Relatedly, multiple levels of change were described for participants. This ranged from shifts in family communication patterns and trust and improvement in symptoms of BN, to insight into their function and broader emotional well-being. These changes were sometimes described differently from the adolescent and the parent/caregiver perspective, but all revolved around a main theme of shifting behaviour, understanding, and relationships. The themes identified in this study add to what is already known about some of the socio-cognitive factors in eating disorders and what might be helpful to target in treatment. For example, the first theme (seeing and being seen) is consistent with previous findings suggesting people with eating disorders value the social interactions of being with others in a group setting [36]. Similarly, the findings of increased family connection and trust reported by participants in theme two (holistic shift) links with suggestions of the importance of treatment needing to address social difficulties which may predispose and maintain eating disorders [25].

Nevertheless, the group identified limits to what was spoken about in the group. Parents/caregivers noted how hard it can be for adolescents with BN to speak about the illness and the associated shame and guilt. Adolescents noted that there were times when they felt the conversation was limited by the group expectations around appropriate information sharing. While the structure was found to be helpful, some expressed a desire to also have some less structured time and to be challenged more.

This last theme really reflected the dialectic between needing the containing nature of a structured group programme to contain anxiety, and the need to be flexible in how treatment is delivered.

The MFT-BN programme is intentionally highly structured as a means of containing group anxiety and helping to promote discussion and cohesion. Given uncertainty and anxiety about the group process is usually highest at the start of treatment, perhaps this structure is only needed early on in process and needs to loosen once participants are more engaged in the process.

The current findings are broadly consistent with previous research on MFT for adolescent eating disorders [19]. This literature highlights the group as place of support and learning associated with symptom change [21–24]. As a point of difference, participants in this study did not mention any unhelpful aspects of the inevitable comparisons that occur between participants in the group. This has been reported by some participants, particularly parents, who receive MFT-AN in other studies [24]. Similarly, participants in MFT-AN studies have not identified wanting to be challenged more in group, as was the case in this study.

This may, in part, reflect differences in the way individuals with BN and their family members present to treatment compared to other eating disorders, such as AN. Additionally, treatment factors may also be contributing to these differences. One important point of difference between MFT-BN and other types of MFT for eating disorders is its intensity. In several MFT-AN models, the group meets for 4 or 5 consecutive full days of intervention [17, 37, 38], which can be followed by additional stand-alone full days of intervention spread over several months [19]. Perhaps participants in the current study felt unchallenged by the group process, at times, simply due to this less intensive format.

The MFT-BN model is intentionally less intensive than those for AN, due the high levels of expressed emotion and criticism often reported within families, as well as the secretive nature of the illness and related emotions of shame and guilt. A briefer, more contained structure with more time devoted to separated adolescent and parent groups is designed to specifically ameliorate some of these factors by ensuring familial patterns are not re-enacted in the group. Feedback that the group may not be challenging enough at times is worth considering in future developments of the model.

### Strengths and limitations

This study has several strengths. It is the first study to explore the qualitative outcomes of MFT-BN for adolescents and adds to the limited overall existing research on interventions for adolescent BN. The sample was also relatively diverse, allowing for a mixture of perspectives. Utilising a combination of focus groups and individual interviews is another strength as it increases data richness.

Regarding limitations, the sample size is small and data collected from only two groups facilitated in one service. The current findings need to be replicated in larger and different groups of young people across a range of treatment settings.

## Conclusions

The current findings support the importance of continuing to develop family, and multi-family, interventions for adolescents with BN. Facilitating group spaces, in which families can learn from each other, appears to be a useful way of increasing support for all, as well as promoting a range of important symptom and relational changes. More data is needed to explore MFT-BN outcomes and experiences across a range of different settings. However, initial data is promising and provides new avenues of exploration in a field that continues to be relatively understudied.

## Data Availability

Data are available from the corresponding author on reasonable request.

## Abbreviations

AN: Anorexia nervosa
BN: Bulimia nervosa
ICD-10: International Classification of Diseases, 10th edition
FT-AN: Anorexia nervosa focussed family therapy
FT-BN: Bulimia nervosa focussed family therapy
MCCAED: Maudsley Centre for Child and Adolescent Eating Disorders
MFT: Multi-family therapy
MFT-AN: Multi-family therapy for anorexia nervosa
MFT-BN: Multi-family therapy for bulimia nervosa
NICE: National Institute for Health and Care Excellence
